# Prevalence and genetic characteristics of *Blastocystis hominis* and *Cystoisospora belli* in HIV/AIDS patients in Guangxi Zhuang Autonomous Region, China

**DOI:** 10.1038/s41598-021-94962-3

**Published:** 2021-08-05

**Authors:** Ning Xu, Zhihua Jiang, Hua Liu, Yanyan Jiang, Zunfu Wang, Dongsheng Zhou, Yujuan Shen, Jianping Cao

**Affiliations:** 1 National Institute of Parasitic Diseases, Chinese Center for Disease Control and Prevention (Chinese Center for Tropical Diseases Research); Key Laboratory of Parasite and Vector Biology, National Health Commission of People’s Republic of China, WHO Collaborating Centre for Tropical Diseases, Shanghai, 200025 China; 2 The School of Global Health, Chinese Center for Tropical Diseases Research, Shanghai Jiao Tong University School of Medicine, Shanghai, 200025 China; 3 Guangxi Zhuang Autonomous Region Center for Disease Control and Prevention, Nanning, 530028 China; 4 Guangxi Medical University, Nanning, 530021 China; 5 Affiliated Hospital of Guilin Medical University, Guilin, 541001 China

**Keywords:** Microbiology, Molecular biology, Diseases

## Abstract

*Blastocystis hominis* and *Cystoisospora belli* are considered to be common opportunistic intestinal protozoa in HIV/AIDS patients. In order to investigate the prevalence and genetic characteristics of *B. hominis* and *C. belli* in HIV/AIDS patients, a total of 285 faecal samples were individually collected from HIV/AIDS patients in Guangxi, China. *B. hominis* and *C. belli* were investigated by amplifying the barcode region of the SSU rRNA gene and the internal transcribed spacer 1 (ITS-1) region of the rRNA gene, respectively. Chi-square test or Fisher’s exact test were conducted to assess the risk factors related to *B. hominis* and *C. belli* infection. The prevalence of *B. hominis* and *C. belli* was 6.0% (17/285) and 1.1% (3/285) respectively. Four genotypes of *B. hominis* were detected, with ST3 (n = 8) and ST1 (n = 6) being predominant, followed by ST6 (n = 2) and ST7 (n = 1). Females had a statistically higher prevalence of *B. hominis* (11.6%) than males (4.2%)*.* The statistical analysis also showed that the prevalence of *B. hominis* was significantly associated with age group and educational level*.* Our study provides convincing evidence for the genetic diversity of *B. hominis*, which indicates its potential zoonotic transmission and is the first report on the molecular characteristics of *C. belli* in HIV/AIDS patients in China.

## Introduction

Globally, HIV infection is an important public health problem. There were approximately 37.6 million people infected with HIV and 1.5 million newly infected worldwide according to the Joint United Nations Programme on HIV/AIDS (UNAIDS) by the end of 2020^1^. In China, the epidemiological status of HIV is alarming, posing tremendous challenges for its control and prevention. It was reported that approximately 9,58,000 people live with HIV by the end of October in 2019 with 1,31,000 new infection from January to October^2^. It is estimated that about 1.1 million people die of AIDS every year, and of which 80% death due to AIDS-related infections^3^. Because of the downregulated immune system of HIV-infected patients, they are highly susceptible to opportunistic pathogens, such as viruses, bacteria, and parasites, of which intestinal parasites are particularly common in HIV-infected patients^4,5^. Diarrhea caused by such intestinal parasites weakens the absorption of antiretroviral drugs and causes nutritional absorption disorders; both exacerbate the condition of HIV/AIDS patients and lower their quality of life^3,6^.

*Blastocystis hominis*, a globally distributed intestinal parasite, was first detected in a human faecal sample in 1911 and mainly parasitizes the large intestine of humans, causing various gastrointestinal symptoms including nausea, abdominal pain, diarrhea, and flatulence, and occasional parenteral symptoms^7,8^. It is estimated that about one billion people in the world are infected with *B. hominis*, whose prevalence is much higher in developing countries (30.0%–100.0%) than in developed countries (1.5%–20.0%)^9–11^. To date, *B. hominis* harbours at least 26 genotypes, of which 10 genotypes, namely ST1–ST9 and ST12, have been found in humans^12–14^. ST3 is reportedly the most common genotype that causes gastrointestinal symptoms^13^. Although the prevalence of *B. hominis* in HIV/AIDS patients has been reported worldwide, the genetic diversity of *B. hominis* in HIV/AIDS patients is rarely documented by empirical data. So far, just three studies of *B. hominis* in HIV/AIDS patients have been carried out in Anhui, Hunan, and Yunnan provinces, China, for which the prevalence of *B. hominis* was 16.2%, 9.9%, and 3.7%, respectively^14^.

*Cystoisospora belli* (formerly *Isospora belli*), originally described by Zaman in 1968^15^, also has a global distribution but it is more common in tropical and subtropical countries, such as India, Nigeria, and Ethiopia^16^. Importantly, *C. belli* has been frequently detected in patients with HIV, Human T-lymphotropic Virus Type I (HLTLV1), Alzheimer’s disease, and colorectal cancer, as well as those having undergone liver and renal transplantation^17–20^. In those HIV/AIDS patients with diarrhea symptoms, the prevalence of *C. belli* could reach up to 20%^21^. Although it occurs worldwide, *C. belli* is a generally overlooked parasite, and one not well studied in China. Hence, further study of this parasite is imperative.

Several cluster studies of *B. hominis* and *C. belli* in HIV/AIDS patients have been conducted, especially in low- and middle-income countries^16,22,23^. But, surprisingly, comparable reports are quite limited for China, especially for *C. belli.* To our best knowledge, the molecular study presented here is the first on this parasite in China.

Guangxi Zhuang Autonomous Region (hereon Guangxi), a provincial-level autonomous region, is located in southern China and borders Vietnam in the southwest. It has a subtropical monsoon climate with abundant rainfall and light. In 2020, the total population of the registered residence in Guangxi was ~ 50 million. It has been reported that the prevalence of HIV/AIDS in Guangxi was 6.6% in 2013, 8.4% in 2014, and 11.2% in 2015, all of which were higher than the corresponding numbers for the general population in China (0.05%)^24,25^. Furthermore, by the end of 2017, Guangxi had reported 124,282 cases of HIV/AIDS, indicating an increase of 78.7% since June 2011 (69,548 cases) and ranking it as second in terms of HIV seropositive cases among the 31 provinces of China^26,27^. Since the Guangxi leads this country in the number of HIV/AIDS patients, investigating their intestinal parasites is of great public health significance^28^. In our previous research, *Microsporidia*, *Cryptosporidium*, and *Giardia* were respectively detected with prevalence of 11.6%, 0.7%, and 2.8% in those participants^5,29,30^. Both *B. hominis* and *C. belli* are recognized as the pathogens that most often cause opportunistic infections in HIV/AIDS patients, posing a prominent threat to the public health^13,16^. Accordingly, the present study aimed to assess the prevalence in addition to genetic characteristics and risk factors of *B. hominis* and *C. belli* in HIV/AIDS patients of Guangxi, China.

## Methods

### Ethics approval and consent to participate

The present study obtained the approval from the Ethics Committee of the National Institute of Parasitic Diseases, Chinese Centre for Disease Control and Prevention (reference no. 2012–12) and all methods were conducted in accordance with the relevant guidelines and regulations as provided in the Declaration of Helsinki. All the patients enrolled in the study were given an oral explanation of the objectives, procedures and potential risks for collection of their faecal samples. Adult participants signed the written informed consent personally. All participants were over the age of 20, so no parental consent was required.

### Sample size calculation

Due to the lack of prevalence of *C. belli* in China, the sample size was calculated based on the prevalence of *B. hominis.* The required sample sized was determined using the formula^31^:$n = \frac{{{\rm Z}_{\alpha }^{2} p\left( {1 - p} \right)}}{{^{{\delta^{2} }} }}$, where $\alpha$ = 0.05, $\delta$ = 0.05, and $p$ = the estimated prevalence of *B. hominis* among HIV/AIDS patients, which was taken as 16.2%^32^. The resulting sample size was 209 patients. Considering that an estimated 10% of the patients might fail to participate in the study, the final sample size was increased to 230 patients. Finally, a total of 285 HIV/AIDS patients registered in nine hospitals in Guangxi were enrolled into the present study.

### Questionnaire, faecal sample collection, and processing

From July 2013 to July 2014, fresh faecal samples (one per patient) were collected, with an in-person, structured questionnaire survey conducted face-to-face to collect the patients’ demographic information (gender, age, education level, occupation), behavioral information (whether drinking boiled water or not, whether receiving HAART treatment or not), HIV transmission route, clinical symptoms (diarrhea), and CD4^+^ cell count.

The collected fresh faecal samples (≥ 200 mg) were first stored at 4 °C with 2.5% potassium dichromate, and then sent to the laboratory of National Institute of Parasitic Diseases, Chinese Centre for Disease Control and Prevention. There, all samples were washed three times with deionized water at 14 000 rpm for 10 min, to remove the potassium dichromate. Then, DNA was extracted from 180 to 220 mg of each faecal sample, by using QIAamp DNA Stool Mini Kit (Qiagen, Hilden, Germany) and following the manufacturer’s instructions. For a higher DNA yield, the lysis temperature was adjusted to 95 °C according to the manufacturer’s recommendation. The final amount of DNA was 200 μl per sample, stored at –30 °C until the PCR analysis.

### Detection of *B. hominis *and *C. belli*

PCR was used to amplify the barcode region of the SSU rRNA gene of *B. hominis*, using primer sets as described by Scicluna^33^, while *C. belli* was detected with the primer sets based on the ITS-1 region of the rRNA gene designed by Reza et al.^17^. All DNA samples were analyzed at least three times. Either *B. hominis*-positive DNA or *C. belli*-positive DNA and nuclease-free water served as the positive and negative controls, respectively. The PCR products (5 μl) were checked by 2% gel electrophoresis, and the products of an expected size (approximately 600 bp for *B. hominis* and 450 bp for *C. belli*) were analyzed using an ABI 3730 DNA Analyzer and Big Dye Terminator v3.1 Cycle Sequencing Kit (Applied Biosystems). ContigExpress program, a component of the Vector NTI Suite 6.0 (https://www.winsite.com/vector /vector + nti/) was used for sequence assembly and wave peak evaluation. The sequences obtained were searched using the BLAST tool (https://blast.ncbi.nlm.nih. gov/Blast.cgi/) in GenBank databases and aligned with representative sequence of *B. hominis* or *C. belli*, respectively.

### Phylogenetic and statistical analysis

Phylogenetic trees for *B. hominis* and *C. belli* were constructed in MEGA 6.0 (http://mega.software.net), based on 1000 bootstrap replicates, using the sequences obtained in this study and representative sequences downloaded from NCBI.

SPSS V20.0 software (https://spss.en.softonic.com/) was used to statistically analyze the data. Chi-square test or Fisher’s exact test was implemented to compare the prevalence between groups classified by gender, age, education level, occupation, whether drinking boiled water or not, whether receiving HAART treatment or not, HIV transmission route, diarrhea, and CD4^+^ cell count. Differences were regarded as statistically significant at *P* < 0.05.

## Results

### Basic information of the study participants

Of the 285 HIV/AIDS patients enrolled, 75.8% (216/285) were males and 24.2% (69/285) were females. Most were farmers (76.1%, 217/285). Patients with a high school and above education accounted for 43.5% (124/285) of the population sample, followed by junior high school (43.2%, 123/285), and then primary school and below (13.3%, 38/285). Of the 285 individuals surveyed, 168 were tested for CD4^+^ cell counts: 29.2% (49/168) had a count ≥ 200, while for the majority (70.8%; 119/168) the count was less than 200. Basic information of this study population appears in Table 1.

**Table 1:** Basic information on participants and assessment of risk factors for *B. hominis* and *C. belli.*

Variable		No. Examined	*Blastocystis hominis*			*Cystoisospora belli*		
			No. Positive (%)	OR^a^ (95% CI)^b^	*χ*^*2*^*/P*- value	No. Positive (%)	OR (95% CI)	*χ*^*2*^*/P*- value
Gender	Male	216	9 (4.2)	3.016 (1.116,8.152)	3.904/0.048	3 (1.4)	0.986 (0.971,1.002)	–/1.000^c^
	Female	69	8 (11.6)			0(0.0)		
Age (years)	< 40	93	1 (1.1)	–^d^	8.443/0.015	0 (0.0)	–	–/0.628^c^
	40–60	113	12 (10.6)			2 (1.8)		
	> 60	79	4 (5.1)			1 (1.3)		
Education level	Primary school and below	38	12 (31.6)	–	51.944/0.000	3 (7.9)	–	–/0.002^c^
	Middle school	123	4 (3.3)			0 (0.0)		
	High school and above	124	1 (0.8)			0 (0.0)		
Occupation	Farmer	217	13 (6.0)	0.981 (0.309, 3.114)	0.000/1.000	3 (1.4)	0.986 (0.971,1.002)	–/1.000^c^
	Others	68	4 (5.9)			0 (0.0)		
Diarrhea	Yes	29	2 (6.9)	0.840 (0.182,3.873)	0.000/1.000	0 (0.0)	1.012 (0.998,1.025)	–/1.000^c^
	No	256	15 (5.9)			3 (1.2)		
CD4^+^ cell count	≥ 200	49	3 (6.1)	0.958 (0.237,3.868)	0.000/1.000	0 (0.0)	1.008 (0.992,1.025)	–/1.000^c^
	< 200	119	7 (5.9)			1 (0.8)		
HIV transmission route	Sex	240	15 (6.3)	0.698 (0.154,3.161)	0.016/0.899	3 (1.3)	0.988 (0.974,1.002)	–/1.000^c^
	Others	45	2 (4.4)			0 (0.0)		
HAART treat	Yes	119	10 (8.4)	0.615 (0.226,1.672)	0.921/0.337	0 (0.0)	1.023 (0.997,1.051)	1.165/0.280
	No	131	7 (5.3)			3 (2.3)		
Boiled water	Yes	266	15 (5.6)	1.969 (0.416,9.322)	0.135/0.713	3 (1.1)	0.989 (0.976,1.001)	–/1.000^c^
	No	19	2 (10.5)			0 (0.0)		

^a^OR Odds ratio.

^b^CI Confidence interval.

^c^Fisher’s exact test.

^d^The “–” symbol indicates the data was not be calculated.

### Prevalence of *B. hominis* and *C. belli*

Overall, the prevalence of these two parasites was 6.7% (19/285); 5.1% (11/216) of males and 11.6% (8/69) of females were positive for at least one of the two protozoan species. Considering *B. hominis*, its general prevalence was 6.0% (17/285), being 4.2% (9/216) in males and 11.6% (8/69) in females. Among these 17 cases of *B. hominis* infection, 10 cases underwent the CD4^+^ cell count test whereas the other 7 did not. The prevalence of *B. hominis* was 5.9% (7/119) in those patients with a CD4^+^ cell count < 200 and 6.1% (3/49) in those with a count ≥ 200. Diarrhea symptoms were self-reported in 2 of the 17 cases of *B. hominis* infection (Table 1).

The prevalence of *C. belli* was 1.1% (3/285), and all the three infected individuals were males. Moreover, co-infection of *C. belli* with *B. hominis* was observed in a farmer aged 50 who had bacterial pneumonia.

### Analysis of risk factors of *B. hominis* and *C. belli*

All 285 participants properly completed the survey questionnaire. They were divided into different groups by gender, age, education level, occupation, whether drinking boiled water or not, whether receiving HAART treatment or not, HIV transmission route, diarrhea, and CD4^+^ cell count. Univariate analysis revealed that three factors were associated with *B. hominis* infection in HIV/AIDS patients: gender (*χ*^2^ = 3.904, *P* = 0.048), age (*χ*^2^ = 8.443, *P* = 0.015), and educational level (*χ*^2^ = 51.944, *P* = 0.000). By contrast, the remaining risk factors tested were not associated with patients’ infection of *B. hominis* (all *P*-values > 0.05; Table 1).

Univariate analysis showed a statistically significant correlation between the prevalence of *C. belli* and different education levels (*P* = 0.002). No significant differences, however, were found between groups formed by other risk factors (all *P*-values > 0.05; Table 1).

### Molecular characteristics of *B. hominis* and *C. belli*

**Figure 1:**
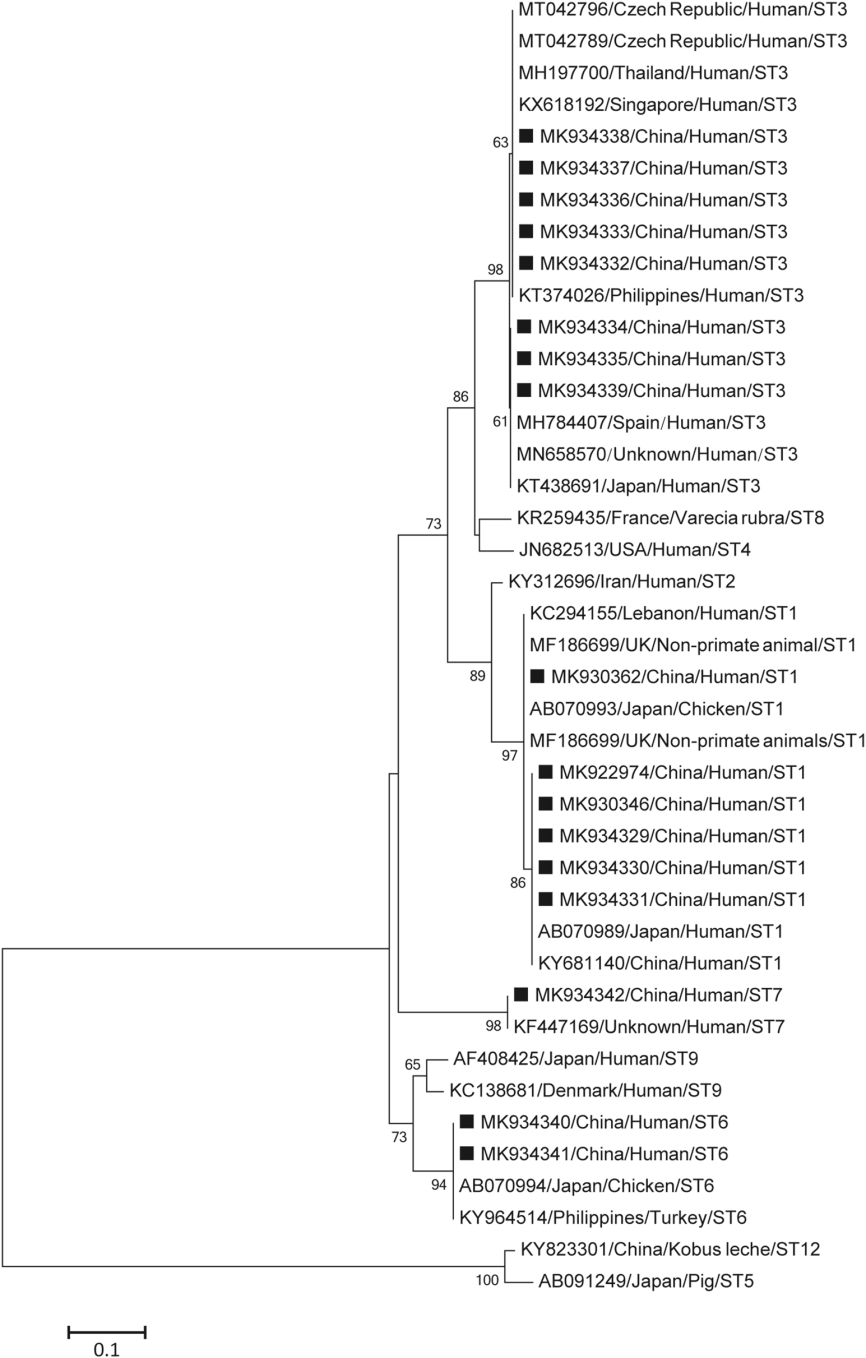
Phylogenetic tree of *Blastocystis hominis* based on the barcode region of the SSU rRNA gene sequence. MEGA 6.0 software (http://www.megasoftware.net/) was used for this analysis, by applying the neighbor-joining distance method with 1000 bootstrap replicates. Individual GenBank accession numbers precede localities, followed by hosts and genotypes. The numbers on the branches are percentage bootstrapping values from 1000 replicates. The scale-bars indicate the number of substitutions per site. Squares indicate *B. hominis* identified from faecal DNA samples in this study.

According to the sequence analysis of 17 *B. hominis* isolates, four genotypes were identified: ST3 (47.0%, n = 8), ST1 (35.3%, n = 6), ST6 (11.8%, n = 2) and ST7 (5.9%, n = 1) (Table 2). Among sequences obtained here, four novel sequences identified as ST1 shared a high identity (99.3%) to the sequence previously identified from chicken (AB070993). The remaining ST1 and ST3 isolates showed 100% homology with other known isolates: namely for ST1, KY681140 (human) and MF186699 (goat); for ST3, KX618192 (human), MH784407 (human), MN658570 human), MT042796 (human), MT042789 (human) and KT438691 (human). The ST6 and ST7 sequences were respectively identical to the known reference sequences KY964514 (turkey) and KF447169 (human) (Fig. 1).

**Table 2:** Genotype distribution of *B. hominis* in the present study.

Region	No. Examined	No. Positive(%)	Genotype			
			ST1	ST3	ST6	ST7
Hospital 1	30	1(3.3)	1	–	–	–
Hospital 2	59	3(5.1)	1	2	–	–
Hospital 3	2	0(0.0)	–	–	–	–
Hospital 4	70	2(2.9)	1	–	–	1
Hospital 5	24	6(25.0)	2	3	1	–
Hospital 6	42	2(4.8)	–	1	1	–
Hospital 7	6	0(0.0)	–	–	–	–
Hospital 8	24	2(8.3)	2	–	–	–
Hospital 9	28	1(3.6)	1	–	–	–
Total	285	17(6.0)	8	6	2	1

**Figure 2:**
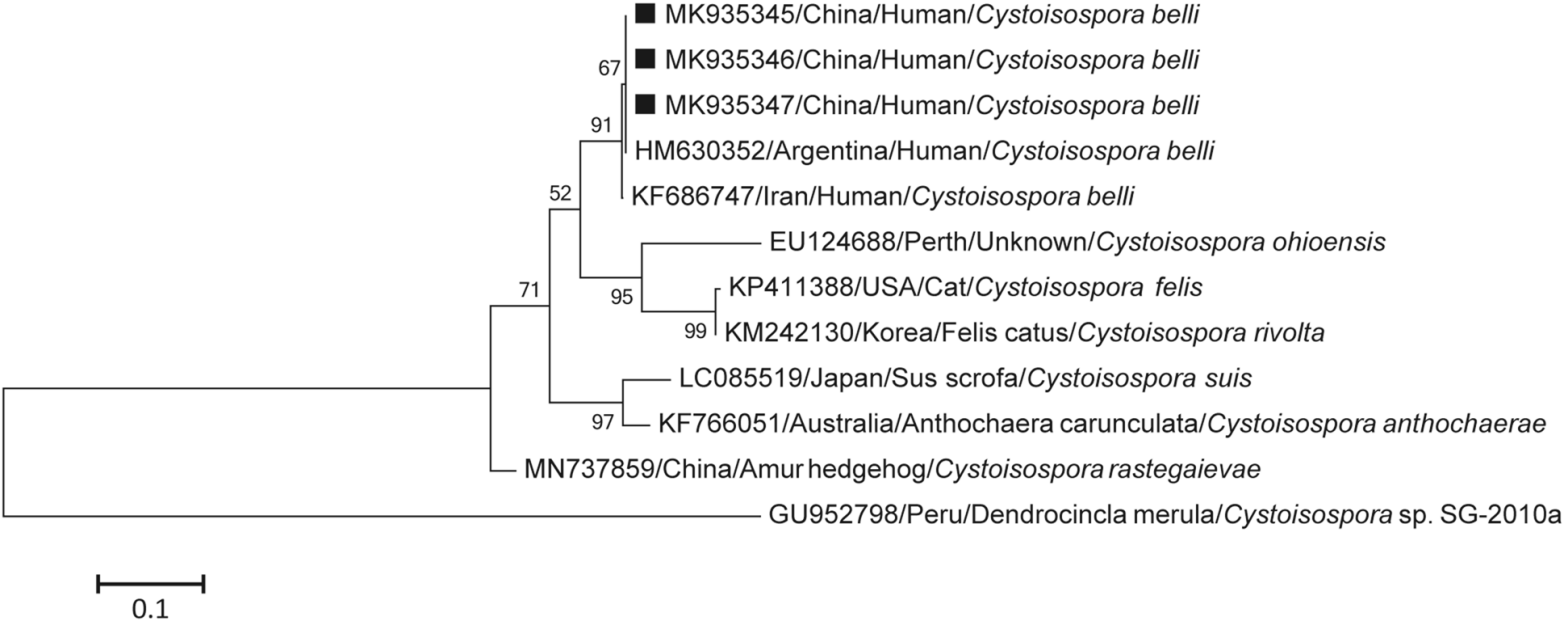
Phylogenetic tree of *Cystoisospora belli* based on the ITS-1 region of rRNA gene sequences. This tree was constructed by the neighbor-joining distance method with 1000 bootstrap replicates, in MEGA 6.0 software (http://www.megasoftware.net/). Individual GenBank accession numbers precede localities, followed by hosts and species. The numbers on the branches are percentage bootstrapping values from 1000 replicates. The scale-bars indicate the number of substitutions per site. Squares represent *C. belli* sequences from this study.

The sequences of the three *C. belli* isolates showed 100% homology with isolates (HM630352) previously identified from an HIV/AIDS patient in Argentina (Fig. 2).

## Discussion

*Blastocystis hominis* and *C. belli* are common, opportunistic intestinal protozoa in immunocompromised individuals, especially in HIV/AIDS patients_._ We determined that the overall prevalence of both species was 6.7% (19/285), with *B. hominis* at least fivefold more prevalent (6.0%, 17/285) than *C. belli* (1.1%, 3/285)*.* As far as we know, this is the first molecular investigation of *B. hominis* in HIV/AIDS patients in Guangxi, China, and of *C. belli* in China.

*Blastocystis hominis* is found globally, but its prevalence in HIV/AIDS patients among countries varies tremendously, from 0.9 to 72.4%^4^. A higher prevalence that reached 88.7% was observed in indigenous children from the Colombian Amazon Basin^34^. Due to the controversial pathogenicity of *B. hominis*, less attention has been paid to it than to *Cryptosporidium*, *Giardia* and *Microsporidia* in China. Nevertheless, a waterborne outbreak of *B. hominis* in China that afflicted 1122 people highlighted the public health significance of this intestinal parasite^35^. Therefore, detection of *B. hominis* is imperative in China.

Generally, few studies of *B. hominis* relying on molecular approaches have been conducted in China. Genotypes of *B. hominis* were analyzed in seven provinces, cities or autonomous regions—Heilongjiang, Guangxi, Yunnan, Zhejiang, Shanghai, Xinjiang and Chongqing—among different population groups, for which its prevalence ranged from 3.7% to 36.6%^14,36–47^(Table 3). We found an occurrence of *B. hominis* in HIV/AIDS patients of 6.0%, lower than that in Fuyang, Anhui Province (16.2%)^48^, yet slightly higher than that in Tengchong, Yunnan Province (3.7%)^14^. In other countries, a higher prevalence of *B. hominis* in HIV/AIDS patients was reported for Peru (24.6%)^49^, Rome (25.0%)^50^, Bogota (25.2%)^51^ and Indonesia (72.4%)^4^, whereas lower values characterized Nepal (0.9%)^52^ and India (3.3%)^53^. Such disparate infection rates may arise from multiple factors, including the research scope, the selected subjects, their living environment as well as local social, economic, and cultural conditions. For example, low socio-economic conditions coupled to a humid and high temperature climate can increase the transmission of *B. hominis*^54^. In addition, diagnostic methods differ in their sensitivity and specificity in detecting *B. hominis.* Molecular approaches based on PCR were shown to be superior to traditional microscopic examination of faecal samples^55^. In southwestern Iran, *B. hominis* occurred in 12.3% and 19.0% of humans according to the Formol-Ether technique and PCR, respectively^56^. In another study of newly arrived immigrants in Qatar, the prevalence of *B. hominis* was 7.6% when assessed by coproscopic methods but much higher (65.2%) by RT-PCR^57^. In other work, in HIV-infected patients, microscopic examination suggested a *B. hominis* prevalence of 21.8% but according to PCR it was 25.0%^50^.

**Table 3:** Genotype distribution of *B. hominis* in different population groups on the basis of geographical locations in China.

Area	Population	Infection rate (%)	Genotype	References
Chongqing	Inpatients	10.6	ST1(2), ST3(8), ST6(10) , ST7(1)	47
Guangxi	College students	–	ST1(4), ST3(17), ST4(4), ST6 (1), ST7(5), Unknown(22)	38
Gunagxi	*B.hominis* isolates	–	ST3(1)	41
Guangxi	*B.hominis* isolates	–	ST1(8),Unknown(2)	42
Heilongjiang	Cancer patients	7.1	ST1(12), ST3(15)	37
Jiangxi	*B.hominis* isolates	–	ST1(13), ST2(2), ST3(14), ST1&ST3(5), Unknown(1)	39
Shanghai	Community residents and patients	1.9	ST1(6), ST2(1), ST3(17), ST6(1) ST1&ST3(2), Unknown(2)	43
Xinjiang	Kindergarten children	14.3	ST1(38), ST2(8), ST3(41)	46
Yunnan	HIV/AIDS patients	3.7	ST1(3), ST3(2), ST4(3), ST7(3), ST12(1)	14
Yunnan	Rural residents	32.6	ST1(16), ST2(1), ST3(55), ST4(1) ST1&ST2(1), ST1&ST3(1), Unknown(3)	36
Yunnan	Diarrhea patients	4.3	ST1(47), ST2(1)	40
Yunnan	Non–diarrhea Patients	10.7	ST1(34)	40
Yunnan	Rural residents	23.7	ST1(38), ST2(7), ST3(93), ST4(1) ST1&ST2(1), ST1&ST3(6), ST2&ST3(1), Unknown(6)	43
Yunnan	Villagers	4.5	ST1(3), ST3(8), ST4(1),Unknown(1)	44
Yunnan	HIV/AIDS patients	3.9	ST1(3), ST3(3), ST4(3), ST7(3)	45
Zhejiang	Inpatients	5.9	ST1(3), ST2(1), ST3(6)	43

In line with the study conducted in Fuyang, Anhui Province, China^48^, we found that women were more likely to get infected with *B. hominis* than men. Differences in nutritional status and access to medical facilities between men and women may be responsible for the increased risk of parasite infection among women^58^. Individuals with an education level of primary schools and below have the highest infection rate of *B. hominis* in Guangxi, hence a limited education and poor awareness of hygiene may contribute to the high infection rate in specific groups. Prevalence in patients grouped by occupation, CD4^+^ cell count, HIV transmission route, and whether they were receiving HAART treatment or not, all showed no statistical differences. Previous studies suggested that people with a CD4^+^ cell count less than 200 are more susceptible to *B. hominis*^56^, and that drinking raw (untreated) water can increase its infection risk^45^. Moreover, three waterborne outbreaks of *B. hominis* have been reported^22,35^, but in the present study the prevalence was similar between boiled water drinkers and raw water drinkers. This result may have two explanations. First, there is no *B. hominis* in the water of this area; second, the ratio of boiled water drinkers was high, accounting for 93.3%.

Currently, the pathogenicity of *B. hominis* remains contentious, as the parasite has been detected in symptomatic and asymptomatic individuals. In the former, diarrhea, abdominal pain, irritable bowel syndrome, constipation, and flatulence are usually reported^7,8^. Mounting evidence shows immunocompromised individuals are more likely to suffer gastrointestinal symptoms related to *B. hominis*. A study performed in cancer patients in China uncovered a significant association between *B. hominis* infection and diarrhea^37^. Similarly, for HIV-infected patients positive association between *B. hominis* infection and flatulence was demonstrated^50^. A variety of clinical symptoms could be related to different *B. hominis* genotypes that can excrete different protease enzymes^59^. For example, Poirier et al. showed that *S*T7 may be responsible for irritable bowel syndrome^60^, while in Iran ST3 may be associated with gastrointestinal disorder^61^. Although ST3 was the predominant genotype identified in our study, no statistical association was observed between *B. hominis* infection and diarrhea.

Presently, *B. hominis* is known to harbour at least 26 genotypes^11,12^. Ten genotypes (ST1–ST9 and ST12) were found in humans, among which ST3 was the most common^37^. The ST1–ST8 have been identified both in humans and various animals, highlighting their capability for zoonotic transmission^12–14^. We know of nine genotypes of *B. hominis* detected so far in HIV/AIDS patients worldwide: ST1–ST7, ST9 and ST12^14,36,45,50,56,62–64^ (Table 4). In our study, four genotypes of *B. hominis* were identified—ST1, ST3, ST6 and ST7—with ST3 the dominant one (47.1%; 8/17), a result consistent with that of studies in HIV/AIDS patients in southwestern Iran and cancer patients in China^37,56^. All the genotypes have zoonotic potential according to the latest research: both ST1 and ST3 are considered the most abundant genotypes ^13,56^ and are widely associated with animals, such as non-human primates, dogs and pigs^65,66^. ST6, detected in indigenous children in the Colombian Amazon Basin^34^, and ST7, detected in HIV/AIDS patients in China’s Yunnan Province and in Iran^14,64^, have been identified from cattle, rodents, birds, chickens, and pigs^65,66^. Additionally, *B. hominis* is viewed as a waterborne pathogen, given its identification in different water bodies including river, wastewater, drinking water, school ponds, and canal water and the three waterborne outbreaks of *B. hominis* infection documented in Italy, China, and Nepal that drew garnered attention to this parasite^22^. Hence, future research should focus on investigating the prevalence and genetic diversity of *B. hominis* in both animals and water in our study area to better understand its transmission dynamics.

**Table 4:** Genotype distribution of *B. hominis* in HIV/AIDS patients worldwide.

Country	No. examined	Infection rate(%)	Genotype	References
China	324	3.7	ST1(3), ST3(2), ST4(3), ST7(3), ST12(1)	14
China	324	32.6	ST1(16), ST2(1) ST3(55), ST4(1), ST1&ST2(1), ST1&ST3(1), Unknown(3)	36
China	311	3.9	ST1(3), ST3(3), ST4(3), ST7(3)	45
Ghana	122	6.6	ST1(4), ST2(2), ST3(2)	62
Iran	268	19.0	ST1(11), ST2(6), ST3(29), ST1&ST3(3), ST1&ST6(2)	56
Iran	1410	3.3	ST3(3), ST4(9), ST5(2), ST7(3),Unknown(5)	64
Malaysia	20	19.8	ST1(2), ST2(1), ST3(9), ST4(6), Unknown(2)	63
Rome	156	25.0	ST1(12), ST2(3),ST3(20), ST4(4)	50

The prevalence of *C. belli* in HIV/AIDS patients varies from country to country, even so within the different areas of the same region^16^. Infection by *C. belli* happens worldwide, especially in tropical and subtropical regions such as India, Iran, Brazil and Ethiopia, but mostly these were sporadic cases or small aggregate outbreaks^16,17,21^. Here, in Guangxi, *C. belli* was detected in three participants, at a prevalence of 1.1%, which is close to that reported for HIV/AIDS patients in Ethiopia (1.3%) ^67^, but lower than that of HIV/AIDS patients in India (16.5%)^68^ and higher than of patients in Burkina Faso (0.7%)^69^. Severe diarrhea due to *C. belli* infection has been reported in immunocompromised patients^17,70^. Contrary to our expectation, the three *C. belli-*infected individuals all reported no diarrhea symptoms. In this study, non-diarrheal individuals accounted for 89.8% (256/285) of the sampled population, which may induce the low prevalence, given that patients with diarrhea often show a high prevalence of *C. belli* (25.0%)^71^.

Here we found that people with a primary school and below education level were more prone to infection by this protist. Yet no statistical link was discernable between its prevalence and the many other risk factors examined. Similarly, a study in Nigeria also could not link isosporiasis to either the gender or age of hosts ^72^.

## Conclusions

The present study revealed the prevalence, genetic characteristics, and risk factors of *B. hominis* and *C. belli* in HIV/AIDS patients in Guangxi, China. Our data indicated the zoonotic transmission of *B. hominis* in this area and relationships between *B. hominis* infection and gender, age, and educational level. Furthermore, our study provided new insight into the molecular characteristics of *C. belli*, providing the first molecular data for this parasite in China. Future studies should be focus on investigating *B. hominis* and *C. belli* in animals and water in the Guangxi area to figure out the sources and routes of these protozoans’ transmission.

## Data Availability

The datasets generated and/or analysed during the current study are not publicly available in order to protect participant confidentiality. The gene sequences identified in this study were submitted to GenBank with accession numbers MK922974, MK930346, MK930362, MK934329-MK934342, and MK935345-MK935347.
